# Molecular dynamics study of the recognition of ATP by nucleic acid aptamers

**DOI:** 10.1093/nar/gkaa428

**Published:** 2020-05-22

**Authors:** Ya-chen Xie, Leif A Eriksson, Ru-bo Zhang

**Affiliations:** School of Chemistry and Chemical Engineering, Beijing Institute of Technology, South Street No. 5, Zhongguancun, Haidian District, 100081 Beijing, China; Department of Chemistry and Molecular Biology, University of Gothenburg, Medicinaregatan 9c, 405 30 Göteborg, Sweden; School of Chemistry and Chemical Engineering, Beijing Institute of Technology, South Street No. 5, Zhongguancun, Haidian District, 100081 Beijing, China

## Abstract

Despite their great success in recognizing small molecules *in vitro*, nucleic acid aptamers are rarely used in clinical settings. This is partially due to the lack of structure-based mechanistic information. In this work, atomistic molecular dynamics simulations are used to study the static and dynamic supramolecular structures relevant to the process of the wild-type (wt) nucleic acid aptamer recognition and binding of ATP. The effects brought about by mutation of key residues in the recognition site are also explored. The simulations reveal that the aptamer displays a high degree of rigidity and is structurally very little affected by the binding of ATP. Interaction energy decomposition shows that dispersion forces from π-stacking between ATP and the G6 and A23 nucleobases in the aptamer binding site plays a more important role in stabilizing the supramolecular complex, compared to hydrogen-bond interaction between ATP and G22. Moreover, metadynamics simulations show that during the association process, water molecules act as essential bridges connecting ATP with G22, which favors the dynamic stability of the complex. The calculations carried out on three mutated aptamer structures confirm the crucial role of the hydrogen bonds and π-stacking interactions for the binding affinity of the ATP nucleic acid aptamer.

## INTRODUCTION

The use of aptamers as hosts to recognize target molecules, especially low weight molecules (1–6), have come to play an essential role in the development of biosensors and detectors, and in drug delivery (7–12). The most common method used to tailor a specific sequence of oligonucleotides or peptides as aptamers is referred to as systematic evolution of ligands by exponential enrichment (SELEX) (13). However, in practice aptamer development is significantly harder than it may appear. Selections can be plagued by a variety of artifacts and complications, including the fact that environmental factors can influence the aptamer structure and thus its binding capacity (14). Nucleic acid aptamers that carry noncanonical tertiary structures has received significant attention in recent years (15–19), as they are thought to exhibit better stability, selectivity and unicity than traditional antigens.

**Figure 1.**
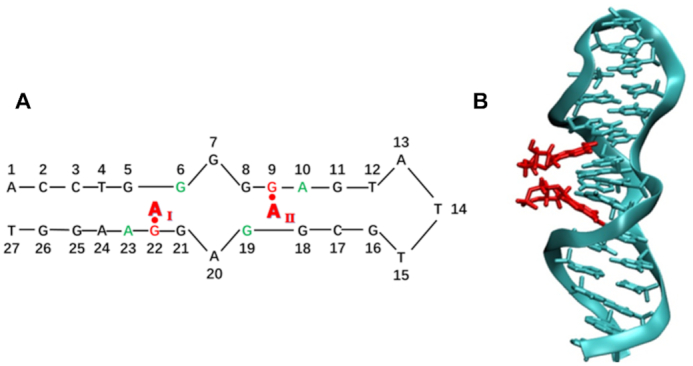
(**A**) The nucleic acid sequence of the wt aptamer-AMP complex. The binding sites of the two bound AMP molecules (A_I_ and A_II_) are highlighted in red and green. (**B**) NMR structure of the AMP_2_–aptamer complex, PDB-ID 1AW4 (22).

One such example is the ATP aptamer, that has been developed and used successfully following the reports of the first wild-type (wt) AMP_2_-aptamer complex (Figure 1) (10,16,17,19,20). It is a 27-mer of single-stranded DNA, and it was initially believed that a G-quartet structure is formed in this aptamer (21). A subsequent coarse NMR structure of the wt aptamer-AMP complex (PDB ID 1AW4) (22) showed that there are two recognition pockets, one being composed of G6, G22 and A23, and the other containing G9, A10 and G19, respectively (Figure 1). Each pocket can accommodate one AMP molecule. When G22 was mutated to A, C or T, the binding affinity to ATP was either reduced or disappeared altogether (21). The effects of the hydrogen bonds between G22 and A of ATP have been addressed earlier (23), indicating that these are highly significant for ATP binding. Our present calculations at the M06-2X/6-31+G(d,p) (24) level show that the hydrogen-bonding interaction between A of ATP and G22 is estimated to be –11.4 kcal mol^−1^. Mutation studies of key bases in the binding region showed that the G6A mutation led to a 20–80% reduction in binding efficiency to ATP (21). Hence, mutant-type (mt) aptamers will to a certain extent retain their capacity to capture ATP, but it is apparent from the above mutation (since G6 does not form hydrogen bonds to ATP) that also other interactions than hydrogen bonds between G22 and ATP are essential. From this perspective, modeling and analysis of the structural details of ATP binding to the aptamer will contribute to the understanding of the recognition mechanism.

From the point of supramolecular assembly, guest-host binding relies on a number of specific interactions between the two molecules. For the recognition of small organic molecules, hydrogen bonds and van der Waals interactions (vdW), generally acting simultaneously, are normally the most important. Absence of aptamer structure information strongly hampers our understanding of, and ability to modulate, the affinity of the aptamer to the targeted molecule on a microscopic scale. In the current work, the wt aptamer–AMP complex based on the 27-mer single-strand DNA, as seen in Figure 1, was used as template. Upon removal of the AMP molecules, the primary wt aptamer–ATP complex was generated through docking. All-atom MD simulations were used to explore the static and dynamic structures and energies during the recognition process. As a comparison, several mutations of key bases in the active site of the aptamer were performed to explore sequence specific effects to the recognition and binding. In order to clarify the potential role of surroundings water molecules, potential of mean force (PMF) calculations were carried out for the supramolecular complexes based on metadynamics and adaptive biasing force methods. These results show that the structure of the active site is essentially unperturbed after insertion of ATP. Compared to the effects of hydrogen bonds, π-stacking interactions are found essential for enhancing the complex stability, which has not been reported before. The previously reported role of hydrogen bonds in the supramolecular complex stabilization are also verified in the current work, through the studies of the mutated systems. Bridging water molecules connecting ATP to the aptamer, are noted to play an important role in regulating the dynamic stability of the complex in the dynamic recognition and dissociation processes.

The current approach provides additional and valuable insights into the recognition process of the nucleotide aptamer towards ATP. From the analyses of the binding interactions, effects of mutations, and role of water, the methodology presented herein appears well suited to assist in the optimization of higher affinity aptamers, also including other substrates, and serve as a tool in the design of new aptamers with different functionalities.

## COMPUTATIONAL METHODS AND DETAILS

The initial wt aptamer-AMP coordinates were extracted from the NMR structure (PDB ID: 1AW4) (22). After removal of the two AMP molecules, the three mutants G6A (mt aptamer 1), A23G (mt aptamer 2), and double mutant G6C/A23C (mt aptamer 3), were generated using Pymol (25). In order to create the ATP-aptamer complexes, AutoDock Vina (26,27) was employed.

To explore the recognition mechanism without interference from a second ATP moiety (ATP being longer and of higher negative charge than the experimentally used AMP molecules), only one active site was used in this study. Thus, each aptamer (wt and mutants) alone and in complex with one ATP molecule were immersed in ca. 4000 TIP3P water molecules (28), respectively, in order to ensure that the systems were completely surrounded by water. The system was neutralized by 0.15 M NaCl to imitate the intracellular environment. For non-bonded interactions, periodic boundary conditions with a cut-off radius of 12 Å were included, and the simulation box size was 43 × 39 × 83 Å. The particle mesh Ewald (PME) algorithm (29) was used to handle electrostatic interactions. Bonds to hydrogen atoms were constrained by the SHAKE algorithm (30). The water molecules were initially minimized by 1000 steps of conjugate gradient with the solute molecule(s) held fixed, followed by 1000 steps of conjugate gradient minimization of the whole system. After a 500 ps heating process from 0 to 298 K in a canonical ensemble (NVT) with the solute fixed, a series of harmonic constrained isothermal-isobaric ensemble (NPT) simulations were performed to enable a controlled release of the solute degrees of freedom. The scaling used for the constraints was 30, 5 and 1 kcal mol^−1^ Å^2^, respectively. Under each constrained scaling, 500 ps MD simulation was carried out using an NPT ensemble. Constant temperature was maintained by the Langevin thermostat method (31) and the pressure was maintained by the Langevin piston Nosé-Hoover method (a combination of the Nosé-Hoover constant pressure method (32) and Langevin dynamics (31)). Last, MD simulations of 150 ns were run for each system in an NPT ensemble with the time step 2.0 fs. For the mt aptamer 3 – ATP complex, two replicas were performed (giving very similar results). The total simulation time in the study is more than 1.3 μs. The trajectory of the last 100 ns of each simulation was used to analyze and display the results using VMD 1.9.3 (33). All MD simulations were performed using the NAMD 2.13 package (34) with the Charmm36 (35) and Charmm36 general force field (CGenFF) (36).

For insights into the ATP recognition or dissociation process, free energy simulations were performed using the meta-eABF (37–39) approach. This method is based on the extended adaptive biasing force (eABF) framework together with metadynamics and has been applied successfully in DNA simulations (40–43). As initial structures for the potential of mean force (PMF) or free energy surface (FES) estimations, the average structures from the MD trajectories were used. Meta-eABF was subsequently run under the NPT ensemble with instantaneous force values accrued in bins 0.1 × 0.1 Å wide. In particular, Gaussian hills with height 0.1 kJ mol^−1^ and width 1.2 Å were deposited. 40 ns meta-eABF simulations were carried out for the four aptamer–ATP complexes.

DFT calculations were performed using Gaussian09 code (44), to assess the interaction of the non-Watson–Crick type of hydrogen-bonding base pairs. Geometry optimizations were performed using the M06-2X/6-31+G(d,p) method (26). The optimized structures were confirmed by frequency calculations at the same level of theory to be real minima with no imaginary vibration frequencies. The M06-2X functional was designed in part to yield more accurate non-covalent interactions containing significant dispersion contributions, as well as reliable thermochemical data (45). The interaction energy reported in the study is defined as Δ*E*_int_ = *E*_complex_ – (*E*_monomer 1_ + *E*_monomer 2_).

## RESULT AND DISCUSSION

### Wild-type aptamer and its complex with ATP

**Figure 2.**
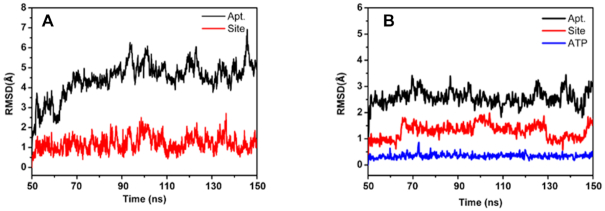
RMSD of (**A**) the wt aptamer and (**B**) the wt aptamer-ATP complex. ‘Site’ is defined as the ATP intercalating region G5, G6, G21, G22, A23.

After 50 ns equilibration simulation of the wt aptamer, root mean square deviation (RMSD) (46,47) was used to monitor the deviation of the structure from the initial position as a measure of system stability. As displayed in Figure 2A, the wt aptamer displays a slight initial fluctuation, but is essentially stable after ∼20 ns production run. The span of the RMSD fluctuations in the interval between 20 and 100 ns of the production run reflects the motile nature of DNA in room temperature (48). G6, G22 and A23 at the active site of the wt aptamer, and their flanking nucleotides G5 and G21 show only minor RMSD fluctuation around 1.2 Å, indicating that the structure of the active site is essentially unperturbed throughout the simulation. In addition, root mean square fluctuations (RMSF) of each base were also calculated and are displayed in Supplementary Figure S1, which shows that the largest fluctuations occur at the terminal bases (1 and 27) and the bases 12–16 in the loop region of the wt aptamer and its ATP bound complex. The fluctuations of the bases G6, A23 and G22, their flanking bases G21 and G5, and A of ATP in the active site are around 1.48 ± 0.28 Å.

**Figure 3.**
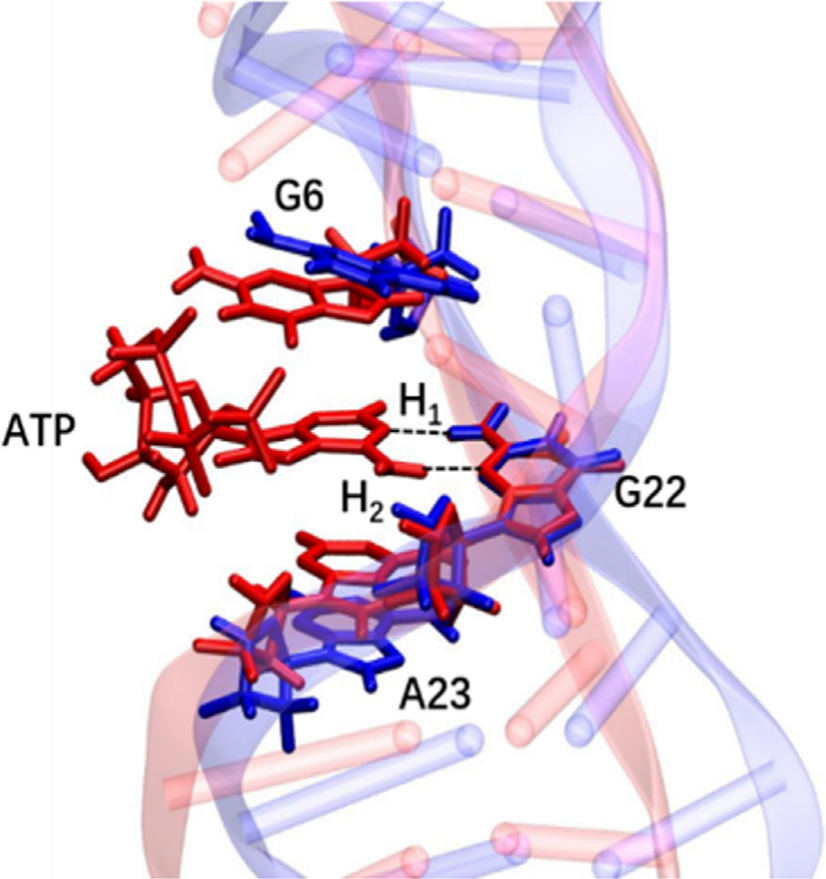
Overlap of the average structures from the MD simulations of the isolated wt aptamer (blue) and its complex with ATP (red). The two hydrogen bonds H_1_ and H_2_ between ATP and G22 are indicated by dashed lines.

Experimentally it was discovered that the aptamer conformation was dependent on ion concentration, and that a higher salt concentration (360 mM NaCl) made the aptamer structure more folded than a low salt concentration (45 mM), which in turn largely promoted the complex formation of the aptamer and ATP. This result illustrates the concept of conformational selection in recognition of the aptamer to ATP (49). In the present studies, 0.15 M NaCl was used to simulate physiological conditions, which hence would favor the stability of the folded conformation of the aptamer. Based on the average structure of the wt aptamer, the wt aptamer–ATP complex structure was generated by docking ATP into the active site as evidenced from the experimental aptamer–AMP structure (22). The RMSD of the MD trajectory of the wt aptamer–ATP complex, shown in Figure 2B, displays a highly rigid structure which indicates that ATP stabilizes the conformation of DNA. The active site of the complex is very little influenced by the dynamics, and ATP displays an almost static structure. Aligning the isolated wt aptamer and the wt aptamer-ATP complex, the two active sites show a good overlap, as displayed in Figure 3. G22 was used as reference for the alignment. For both A23 and G6, ATP appears to pull these closer via π-stacking interaction formed upon the intercalation. Combined with the RMSD plots of the active site, it can be concluded that the structure of the binding site is very rigid, which contributes to the recognition of ATP by the wt aptamer in a key-in-lock mechanism.

**Figure 4.**
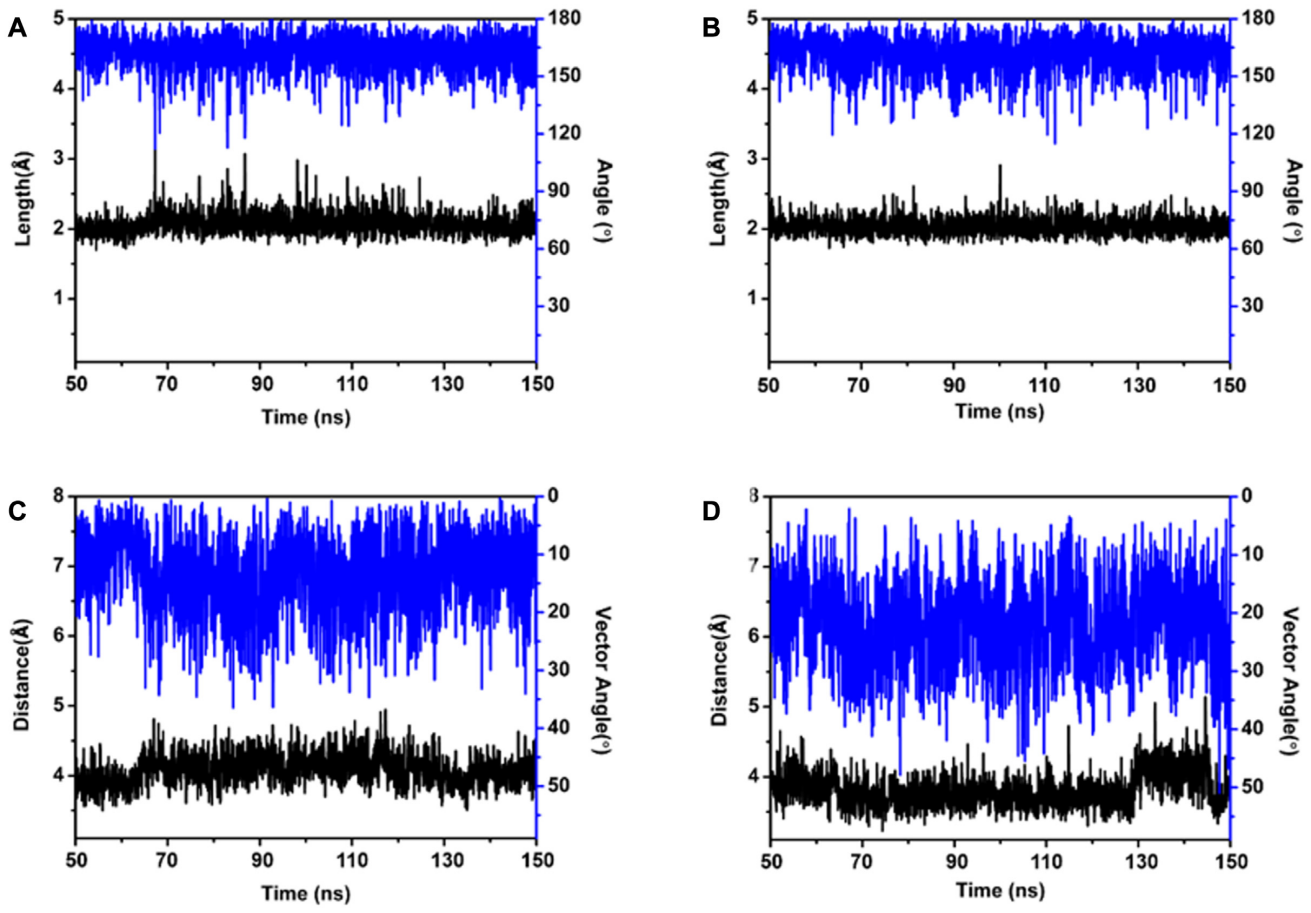
Data for the hydrogen bonds (**A**) H1 and (**B**) H2, of the wt aptamer-ATP complex, respectively. Length in black (left scale) and angle in blue (right scale). Distance (black, left scale) and vector angle (blue, right scale) between (**C**) G6 and ATP and (**D**) ATP and A23. Distances and angles as defined in the text.

In the binding motif, we observe the two hydrogen bonds between ATP and G22 (ATP:H61$\cdots$N3: G22 and ATP:N1$\cdots$H22:G22, defined as H_1_ and H_2_, respectively, in Figure 3), which is consistent with the observations in the NMR experiments (22). Hence, these hydrogen bonds can easily be identified as key interactions, and were thought to be crucial factors for the aptamer binding to ATP (10). The stability of the hydrogen bonds were analyzed herein by plotting the length and angle vs MD simulation time. As shown in Figure 4, the lengths of the two hydrogen bonds are 2.06 ± 0.16 Å and 2.04 ± 0.13 Å, and the angles are calculated to be 162.0 ± 9.5° and 161.3 ± 9.9° (Figure 4A and B), respectively, and are essentially unchanged throughout the simulations. A transient hydrogen bond is also seen between an oxygen of the triphosphate group of ATP and the NH2 group of G21, that might be assisting during the association process between ATP and the aptamer. In addition, the adenine of ATP clearly forms π-stacking interaction with G6 and A23, respectively.

**Figure 5.**
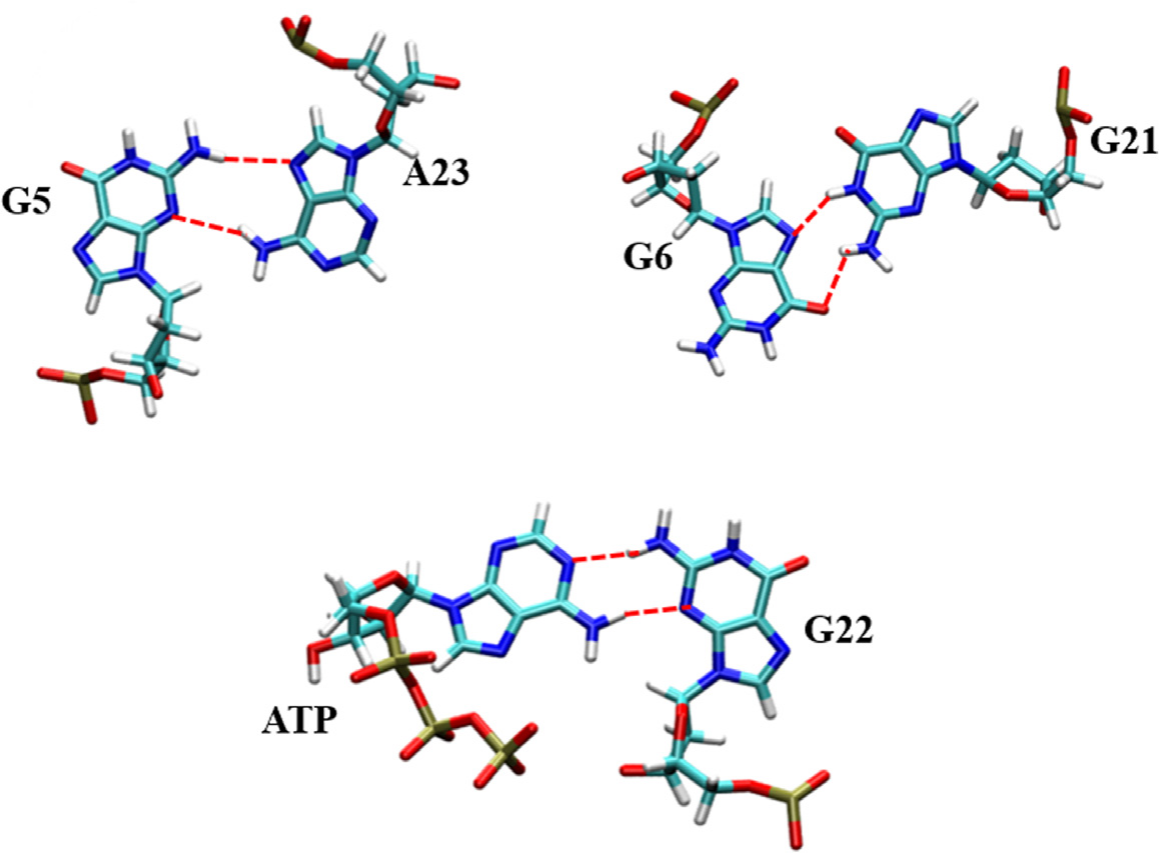
Average structures of the significant non-Watson–Crick base pairs in the wt aptamer, which preserve the stability of the active site. Hydrogen bonds are indicated with dashed lines.

G6 and A23 can be observed to form noncanonical hydrogen bonds with G21 and G5, respectively, as displayed in Figure 5. These interactions were also maintained during the MD simulations, showing that the positioning of A23 and G6 are well conserved in the wt aptamer structure. For insights into the molecular details of the binding site, the π-π interaction arising from the stacking of A23, Adenine of ATP and G6 were analyzed in terms of the centroid–centroid distance and the angle of the vectors of the two adjacent base planes, and are displayed in Figure 4C and D. For G6 and ATP, the distance between the π domains of guanine and adenine is 4.09 ± 0.22 Å, and the average vector angle is 13.4 ± 6.9°, which shows that the two bases are almost parallel to each other. In contrast, the π domains of the adenines of A23 and ATP display a shorter distance (3.80 ± 0.24 Å) and larger vector angle (22.0 ± 7.8°).

**Figure 6.**
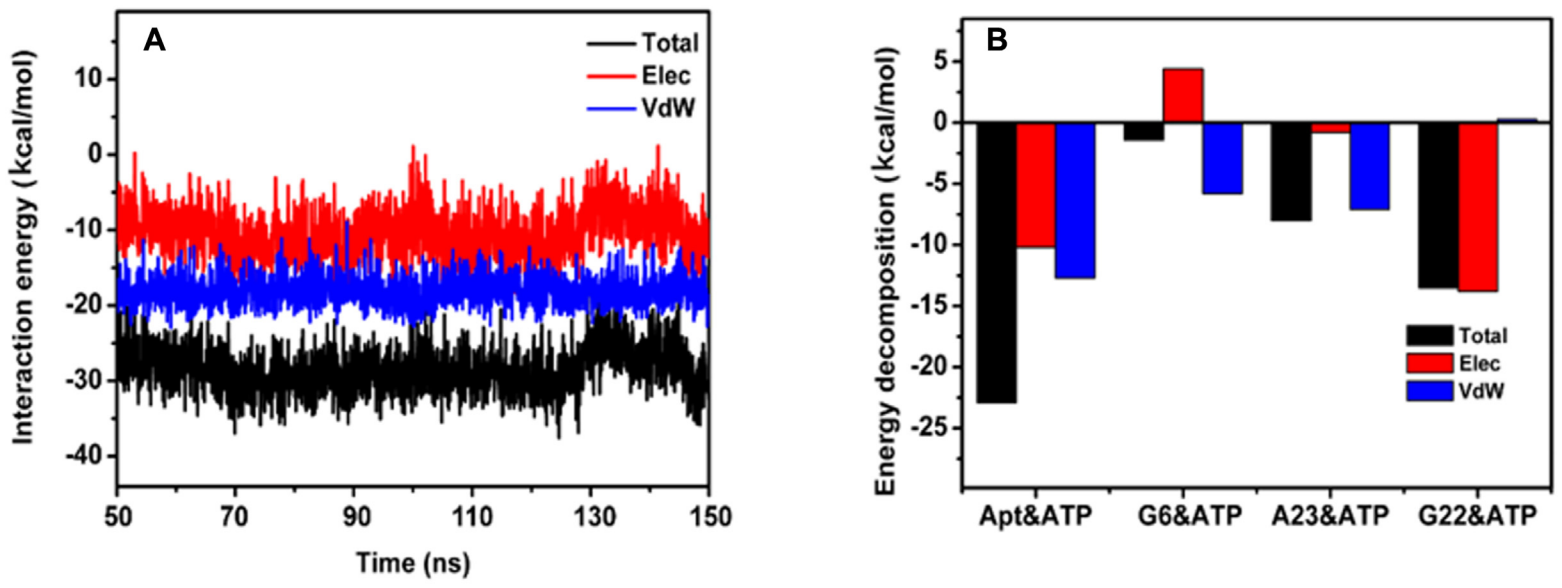
Total electrostatic and van der Waals interaction energy (**A**), and contribution of the three active site bases and their individual components to the binding energy (**B**), for the wt aptamer–ATP complex.

In order to further explore the effects of the non-covalent interactions on the affinity, an interaction energy decomposition analysis was performed based on the MD simulation data. As shown in Figure 6a, the average interaction energy between ATP and the wt aptamer is –28.6 ± 2.9 kcal mol^−1^. The contribution from the vdW interaction is –18.1 ± 1.9 kcal mol^−1^, which is stronger than the electrostatic interaction, –10.5 ± 3.3 kcal mol^−1^. Thus, the vdW interaction should be the main driving force for the wt aptamer recognition of ATP. In addition, the contribution from each nucleobase in the active site was addressed (Figure 6b). The vdW energy contribution from the aptamer active site is -12.2±2.6 kcal mol^−1^, which arises from the interaction with G6 and A23 in almost equal contribution. The relative electrostatic energy contribution is +4.5 ± 1.6 kcal mol^−1^ from these same bases. These results show that the π−π base stacking interaction largely contributes to the total vdW energy (–18.1 ± 1.9 kcal mol^−1^). Electrostatic interaction between G22 and A of ATP, that was previously thought of as a key force to formation of the complex, is –12.7 ± 3.3 kcal mol^−1^, which is very close to our computed value (–11.4 kcal mol^−1^) using the M06-2X/6-31+G(d,p) method. Hence, we conclude that both vdW and electrostatic energy contribution from the nucleosides of the active site are driving forces to stabilize the formed wt aptamer–ATP complex. We conclude from this analysis that ATP is also stabilized within the wt aptamer by significant π–π interaction, in addition to the previously addressed hydrogen bonds.

The interaction energy of the triphosphate unit of ATP with the wt aptamer was analyzed and is shown in Supplementary Figure S2. As expected, vdW interactions are negligible for the complex, and the electrostatic interaction between the wt aptamer and the triphosphate is highly repulsive since they both carry negative charges. However, the negative charges of the triphosphate unit should be screened by sodium ions in the solution. From the MD simulations, there are on average three sodium ions around the triphosphate. The interaction energy between the triphosphate together with three sodium ions and the wt aptamer is –10.9 ± 7.9 kcal mol^−1^, as shown in Supplementary Figure S2B. In conclusion, the negative charge of the triphosphate in ATP is favorable for the association with the DNA aptamer *if* the concentration of sodium ions is high enough. In our studies, the concentration of 0.15 M NaCl was used based on normal physiological conditions.

The contribution from the sugar unit of ATP to the interaction with the aptamer was also analysed (Supplementary Figure S2C). Again, the contribution is largely electrostatic, with an energy of ∼–40 kcal/mol.

### Analysis of key bases in the mutant systems

Based on the structural and energetic analysis of ATP binding to the active site of the wt aptamer, G6 and A23 were identified to play a key role in the binding. Further exploration of the roles of these bases was conducted by analyzing the mutants G6A, A23G and G6C/A23C, referred to as mt aptamer 1, 2 and 3, respectively. The same simulations as above were performed for the mt aptamers and their complexes with ATP.

**Figure 7.**
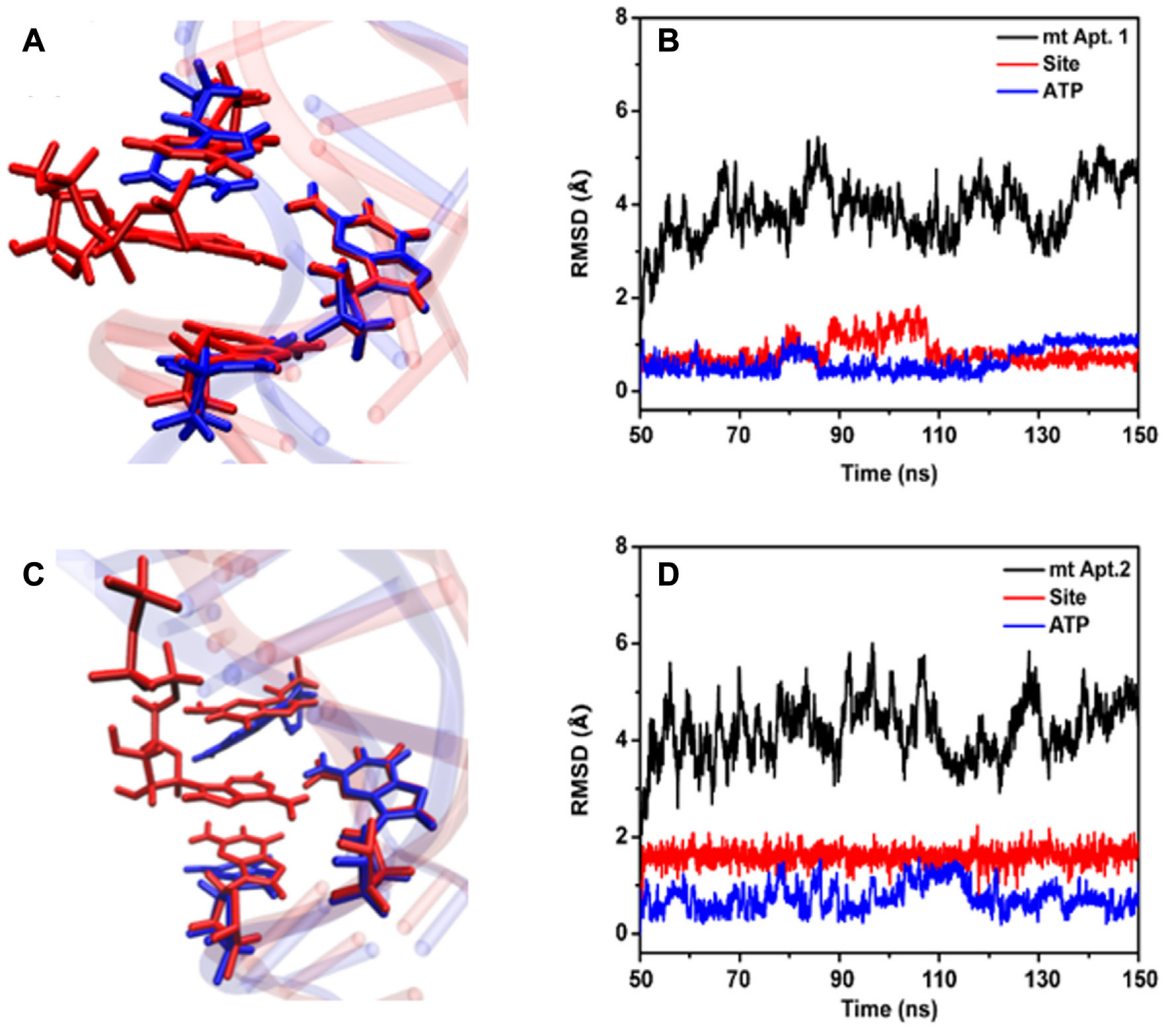
(**A**) Overlap between mt aptamer 1-ATP complex (red) and isolated mt aptamer 1 (blue) after MD simulation. (**B**) RMSD of the mt aptamer 1-ATP complex. (**C**) and (**D**): Same as (A) and (B) for the mt aptamer 2-ATP complex.

For the unbound mutant aptamers 2 and 3, the RMSD increase throughout the simulations, but the binding site remains relatively stable for all mutants (Supplementary Figure S3). The RMSF data for mt aptamers 1 and 2 (Supplementary Figure S1) reveal that besides the large values for the terminal bases and the loop region as mentioned above, there are also increased movements of the bases neighbouring the binding site as compared to the wt system. In Figure 7, we show the average structures of mt aptamers 1 and 2 with/without ATP bound from the MD production simulations (Figure 7A and C), and the corresponding RMSD plots of the mt aptamers 1 and 2 in complex with ATP (Figure 7B and D). Comparing with the wt aptamer structure in Figure 3, the average structures of the mt aptamers also display well defined binding regions. For mt aptamer 1, the aptamer and its active site are largely inflexible during the simulation, as reflected in the small fluctuation in RMSD values. In contrast, the RMSD fluctuation of mt aptamers 2 and, in particular, 3 are larger, albeit the structures of the binding regions are largely retained (Figure 7 and Supplementary Figure S3). In the binding motifs (Supplementary Figure S4), A of ATP, G6/A6 and A23/G23 form similar hydrogen bonds with G22, G21 and G5, respectively, in the wt aptamer, mt aptamer 1 and mt aptamer 2 complexes with ATP. In contrast, in the mt aptamer 3 complex with ATP, A of ATP forms two hydrogen bonds with G21 instead of G22, and no bases form hydrogen bonds with C6 or C23 providing increased flexibility in this system.

The key structural parameters for the active site and ATP in the wt and mutant complexes are shown in Table 1. In the mt aptamer 1-ATP complex (G6A), A6, A23 and A of ATP keep a perfect π-stacking, as evidenced by the measured face-to-face distances and intersection angles. The hydrogen-bonding interaction between G22 and A of ATP, is, however, slightly weakened as seen in the longer N–H···N distances and the smaller bonding angles when compared to those in the wt aptamer–ATP complex. In the mt aptamer 2–ATP complex (A23G), larger intersection angles and face-to-face distances are found, but hydrogen bond H2 is the shortest of all complexes (Table 1). As discussed above, in the binding state of the mt aptamer 3-ATP complex (G6C/A23C), finally, hydrogen bonds are formed between G21 and ATP, but not between G22 and ATP. Instead, access to G22 is blocked by C23 and only initial π–π interaction is observed between A of ATP and C6/C23. Although the structure of the mt aptamer 3–ATP complex was maintained for up to ∼105 and 127 ns of the two MD simulation replicas, the large fluctuation in RMSD values reveals that this complex is not stable (Supplementary Figure S5). Even for the most stable of the two simulated complexes, the π–π interaction between ATP and C23, C6 was only retained for about 50 and 90 ns, respectively (Supplementary Figure S6). Loss of the π-π interaction is followed by influx of water molecules, which contributes to disrupt the hydrogen bonds between ATP and G21. Thus, bases 6 and 23 seem to play a vital role in ‘pinching’ ATP and thereby block the influx of water molecules. Upon release of ATP, the energy of mt aptamer 3 decreases, which again shows that the binding of mt aptamer 3 to ATP is not preferred (Supplementary Figure S5). Hence, besides the hydrogen bonds between G22 and A of ATP, the π–π interaction between bases 6, 23 and A of ATP appears to be required for the formation of a stable aptamer–ligand complex.

**Table 1.** Key structural parameters (distances in Å and angles in degrees) of all complexes studied. Standard deviations given in parenthesis

	H-bonds				π-stacking			
	H1		H2		ATP & base 6		ATP & base 23	
Complex system	Length	Angle	Length	Angle	Distance	Angle	Distance	Angle
wt apt.	2.07	162.0	2.03	161.4	4.09	13.4	3.80	22.0
	(±0.16)	(±9.5)	(±0.13)	(±9.9)	(±0.22)	(±6.9)	(±0.24)	(±7.8)
mt apt. 1 (G6A)	2.12	154.9	2.15	151.0)	3.75	7.2	3.69	6.5
	(±0.18)	(±11.9)	(±0.39)	(±19.3)	(±0.18)	(±5.4)	(±0.19)	(±5.3)
mt apt. 2 (A23G)	2.11	159.4	1.84	172.6	4.68	20.2	4.05	22.2
	(±0.15)	(±10.5)	(±0.15)	(±8.6)	(±0.21)	(±7.5)	(±0.19)	(±4.7)
mt apt. 3^a^ (G6C/A23C)	/	/	/	/	4.04	13.3	3.79	10.9
					(±0.18)	(±5.4)	(±0.38)	(±7.4)

^a^Data for replicate 1 shown here, prior to dissociation. Similar values were seen in replicate 2.

The binding energy of mt aptamer 1 with ATP is estimated to be –23.1 ± 2.3 kcal mol^−1^, and –18.9 ± 1.9 kcal mol^−1^ for the mt aptamer 2–ATP complex (Table 2). These are both smaller than that of the wt aptamer–ATP complex (–28.6 ± 2.9 kcal mol^−1^). The stability ordering is thus in good agreement with the experiment results (21). It is worth noting, however, that the interaction energy of the mt aptamer 1-ATP complex is relatively close to that of the wt aptamer–ATP complex, which also matches the experimental results (21).

**Table 2.** Binding energy decomposition analysis (kcal mol^−1^) for the contributions from the key bases in the mutant complexes 1 and 2. Standard deviations given in parenthesis

	mt aptamer 1 (G6A)				mt aptamer 2 (A23G)			
Interaction energy	A6	A23	G22	Aptamer	G6	G23	G22	Aptamer
Elec	5.9	−1.3	−14.1	−8.5	−0.9	0.5	−14.1	−10.6
	(±1.4)	(±1.1)	(±2.7)	(±2.9)	(±1.5)	(±2.0)	(±1.3)	(±2.5)
VdW	−7.4	−8.0	−0.8	−14.6	−3.1	−7.4	1.4	−8.3
	(±0.83)	(±0.7)	(±1.3)	(±1.8)	(±0.7)	(±1.4)	(±0.6)	(±1.7)
Total	−1.5	−9.3	−14.9	−23.1	−4.0	−6.9	−12.7	−18.9
	(±1.4)	(±1.4)	(±2.0)	(±2.3)	(±1.3)	(±1.4)	(±1.4)	(±1.9)

### Binding free energy calculation

Understanding the dynamic process of the wt aptamer affinity to ATP can provide further insights into the recognition mechanism of the nucleic acid aptamer. To address this issue, free-energy profiles were computed for the wt and mt aptamer-ATP complexes using a progressive sampling algorithm, meta-eABF (37). In short, the center-of-mass (COM) separation between the ATP molecule and G22 was considered as a collective variable (CV), which represents the transition of ATP from the binding cavity to its free state. Moreover, in order to increase the efficiency of the sampling, the coordinates of the involved atoms of the aptamer active site and ATP were described by a spherical coordinate system (50) centered on the adenine of ATP. During the dissociation progress, the water molecules in the active region are of particular interest.

**Figure 8.**
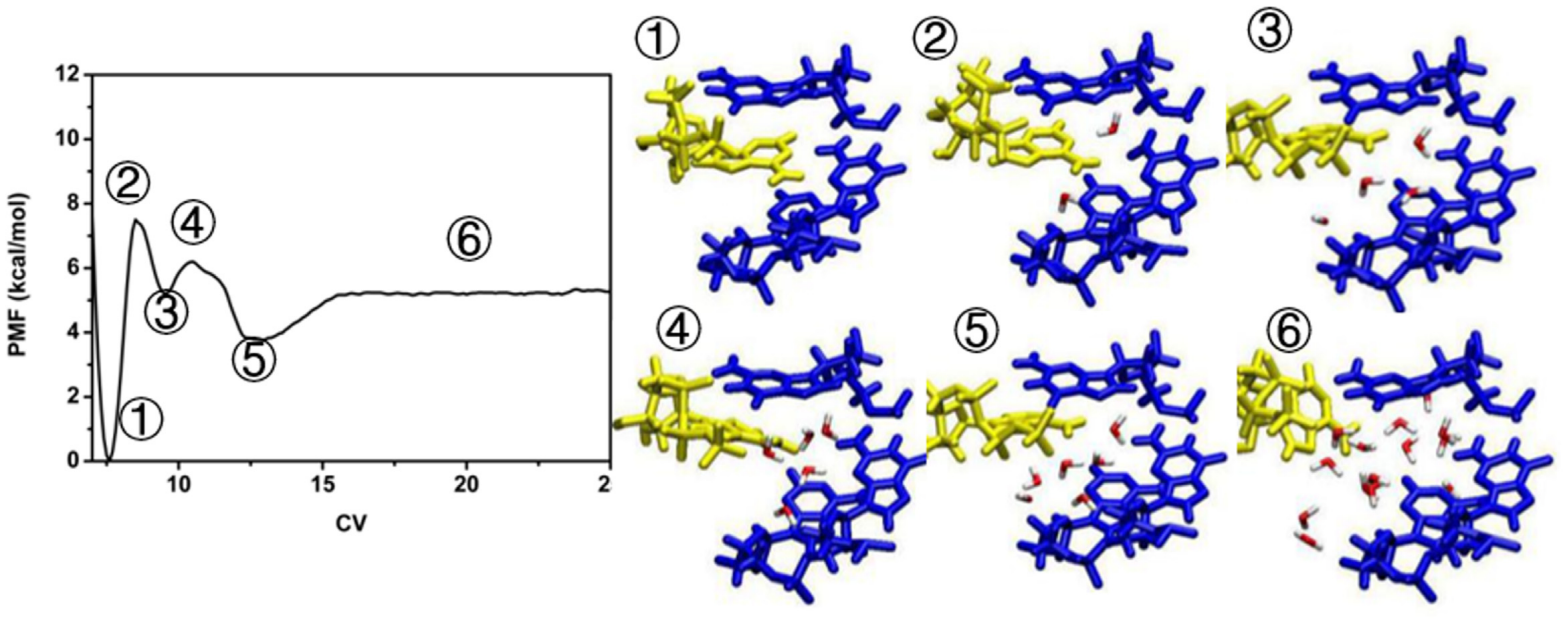
Binding free energetic profile of the wt aptamer - ATP complex. Snapshots 
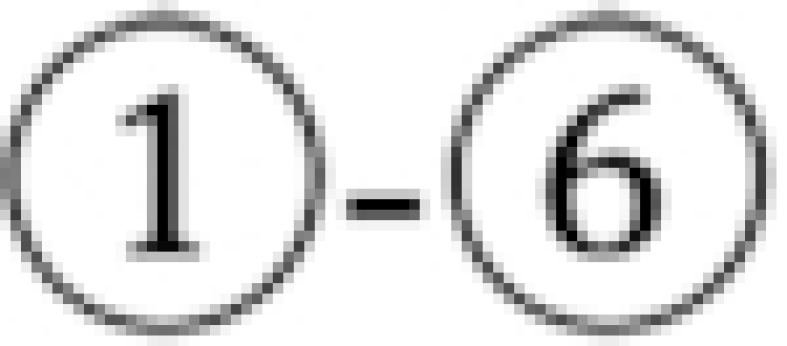
illustrate the main structural changes of ATP (yellow) and wt aptamer (blue) during the 40 ns meta-eABF simulation.

For the wt system, we examined the free energy surfaces (FES) for the dissociation process during 1, 5, 10, 20, 25, 30 and 40 ns simulation time, respectively (Supplementary Figure S7). For simulation times less than 25 ns, the free energy surfaces were not converging properly. For the trajectory time of 25 ns, the peak relative to a COM separation of 12 Å shows a free energy barrier of 8.5 kcal mol^−1^, which is only slightly higher than the values obtained using 30 or 40 ns trajectories. The FES from the 40 ns simulation is very similar to that of the 30 ns simulation, and we thus consider the 40 ns simulations as sufficiently converged to describe the dissociation progress (Supplementary Figure S8). At the lowest point along the PMF curve ((1) of Figure 8), the wt aptamer and ATP are bound by hydrogen bonds and π-stacking interactions, and no water molecule is observed in the recognition region. From this point, an energy barrier 7.4 kcal mol^−1^ ((2) of Figure 8) must be overcome to break the hydrogen bond between G22 and ATP; comparable to the 8.5 kcal mol^−1^ barrier obtained from the 25 ns simulation trajectory. At this point, there is one water molecule forming hydrogen-bonded bridge connecting G22 with ATP. The π-stacking between ATP, G6 and A23 is retained during the process.

After breaking the hydrogen bonds, a small basin ((3) in Figure 8) is found in the FES. As the COM distance increases, a second water enters between ATP and G22 with a very small barrier of 1.0 kcal mol^−1^ (point (4) in Figure 8), which further weakens the interaction between ATP and the aptamer. As an increasing number of water molecules penetrate into the binding cavity, ATP dissociates from the active region, the π-stacking is completely broken, and the PMF curve levels out at point (6) in Figure 8. From this point, further changes in interaction between ATP and the wt aptamer are negligible. The binding free energy of the wild-type aptamer to ATP is –5.4 kcal mol^−1^, which is consistent with the –6.7 kcal mol^−1^ reported from previous experiments (19).

Compared to the dissociation/recognition process of the wt aptamer-ATP complex, the mutated aptamer 1 and 2 systems perform very similarly. However, the barrier heights from breaking the hydrogen bonds between the G22 and ATP are notably smaller than in the wt aptamer-ATP complex (Supplementary Figure S9), in particular for the G6A mutant (mt aptamer 1). Moreover, the basin (5) of Figure 8 in the wt aptamer–ATP complex shows that water molecules in the active region significantly influence the dynamic stability of the complex in the dissociation/recognition processes. A corresponding narrow and shallow basin before the complete dissociation is found in the mt aptamer 1–ATP system, which reflects high dynamic instability. In relative terms, the mt aptamer 2-ATP system displays higher dynamic stability than the mt aptamer 1–ATP system. The two mt aptamer–ATP complexes have almost the same binding free energies, –4.5 and –3.4 kcal mol^−1^, respectively, which is slightly smaller than that of the wt aptamer. Taken together, the present MD studies provide clear picture of the stronger interactions provided by the wt aptamer in recognizing and binding to ATP, in agreement with the experimental results (19,21).

## CONCLUSION

Based on the current MD simulations of the wt and mt nucleic acid aptamers of the biologically important molecule ATP, a binding mechanism is proposed. The vdW energy between the wt aptamer and ATP is clearly larger than the electrostatic interaction, showing that the vdW energy plays a more prominent role than previously described, and have an at least as important role as the hydrogen bonds formed to G22. Additional calculations show that G6 and A23 engage in π-π interaction with the adenine ring system in ATP, which provides a large contribution to the total vdW energies. By extensive MD simulations based on the meta-eABF method, the binding free energy is estimated to be –5.4 kcal mol^−1^ for the wt aptamer–ATP complex, which is consistent with the experimental value –6.7 kcal mol^−1^ (19). In the dissociation/ recognition process of the complex, bridging water molecules connecting ATP with the aptamer are seen to regulate the dynamic stability of the complex. Three mutant aptamers are introduced to highlight the role of the G6 and A23 bases involved in π−π interactions to ATP. From the present computations it can be concluded that the π-stacking between G6, A23 and A of ATP is very important for the stability of the wt aptamer–ATP complex, in addition to the hydrogen bonds to G22. Based on the association and recognition mechanisms of the nucleic acid aptamer to ATP, the present study can form a starting point to optimize the design of new ATP aptamers with higher affinity, or to tailor nucleotide aptamers for other substrates.

## DATA AVAILABILITY

Simulation protocols, input files and result files are freely available at zenodo.org as DOI: 10.5281/zenodo.3667590.

## Supplementary Material

gkaa428_Supplemental_File
