# Supplementary material for: Molecular dynamics study of the recognition of ATP by nucleic acid aptamers
Source: Nucleic Acids Res. 2020 May 22;48(12):6471–80. doi: 10.1093/nar/gkaa428 (PMC7337527; doi:10.1093/nar/gkaa428)
Supplement: gkaa428_Supplemental_File [file gkaa428_supplemental_file.pdf]

Electronic supplementary information

Molecular Dynamics study of the Recognition of ATP by Nucleic Acid Aptamers

Ya-chen Xie[a], Leif A. Eriksson[b,*] and Ru-bo Zhang[a,*]

[a] School of Chemistry and Chemical Engineering, Beijing Institute of Technology, South Street No. 5, Zhongguancun, Haidian District, 100081 Beijing, China.

[b] Department of Chemistry and Molecular Biology, University of Gothenburg, Medicinaregatan 9c, 405 30 Göteborg, Sweden

[*]Corresponding authors:

zhangrubo@bit.edu.cn; leif.eriksson@chem.gu.se

FIGURE **PAGE**

S1. RMSF curves for bound and unbound wt, mt1 and mt2 aptamers. 2

S2. Interaction energy curves of triphosphate and sugar units of ATP with the wt aptamer. 2

S3. RMSD curves from MD simulations of for unbound aptamers. 3

S4. Average structures of the significant non-Watson-Crick base pairs in the aptamers and their complexes with ATP 3

S5. RMSD curves and base interaction sin the mt aptamer 3 – ATP system. 4

S6. Hydrogen bond and π-stacking distances and angles for the most stable mt aptamer 3 – ATP replica. 4

S7. Free energy profiles as a function of the CV COM distance between G22 and ATP in the wt aptamer – ATP complex 5

S8. Time evaluation plot of the examined CV of the meta-eABF simulations of the aptamer complexes. 6

S9. PMF curves for the separation of ATP and mutant aptamers at different reaction times. 7

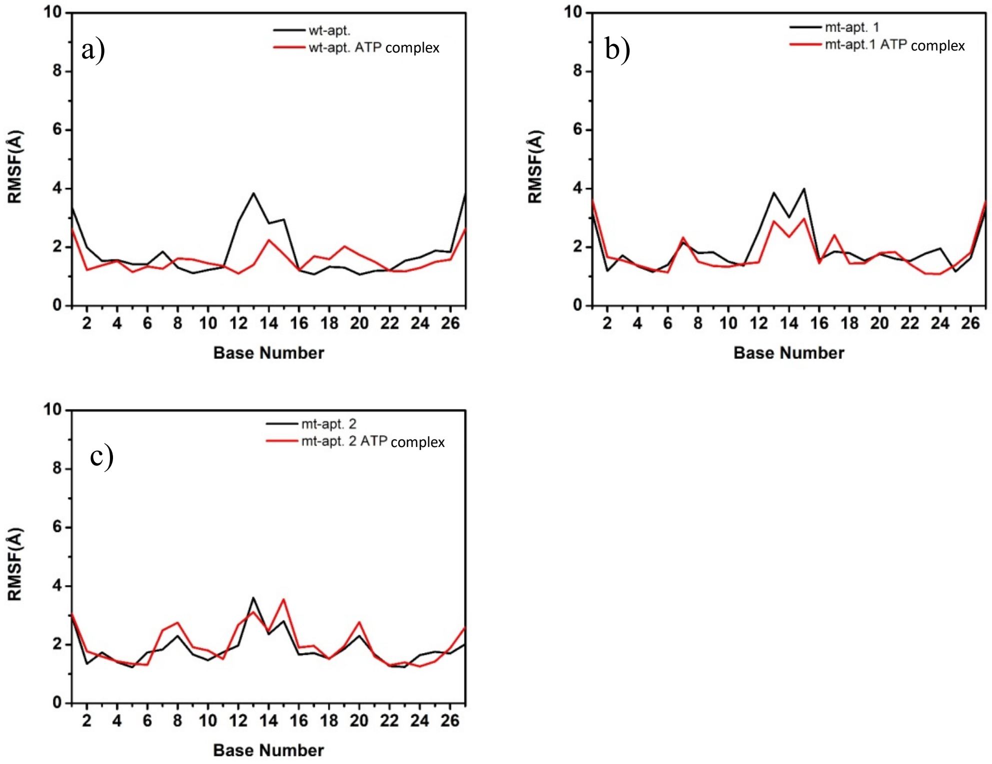

Fig. S1 RMSF for a) wt apt. and ATP-aptamer complex; b) mt apt.1 and ATP-mt apt. 1 complex; c) mt apt.2 and ATP-mt apt.2 complex. Black line shows the unbound aptamer and red line the complex.

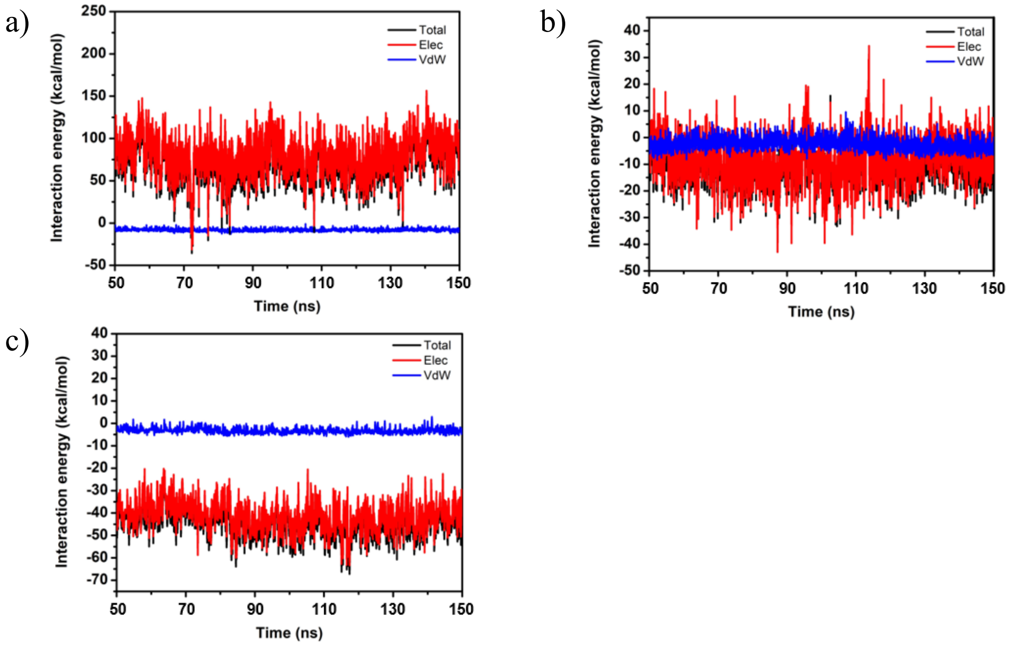

Fig. S2 a) The interaction energy of wt-aptamer and the triphosphate of ATP; b) the interaction energy of triphosphate (with three sodium) and aptamer; c) interaction energy of the sugar moiety of ATP with the wt aptamer.

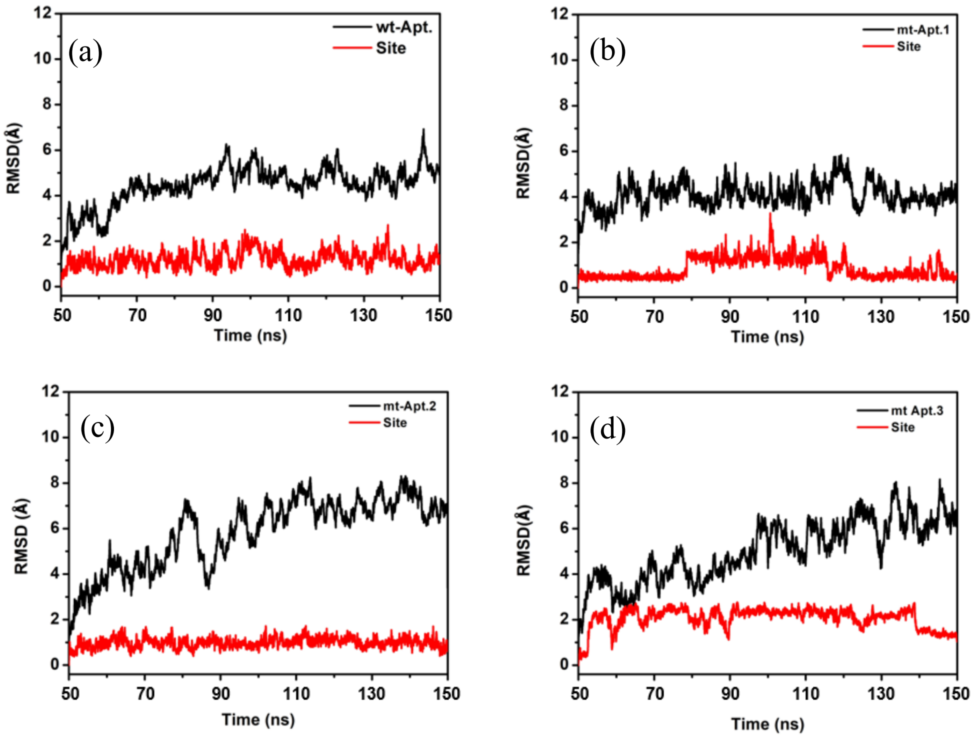

Fig. S3 RMSD of (a) wt aptamer, (b) mt 1 apt., (c) mt 2 apt. and (d) mt 3 apt. 'Site' is defined as the ATP intercalating region, including bases 6, 23 and 22 (21 in mt3 apt.).

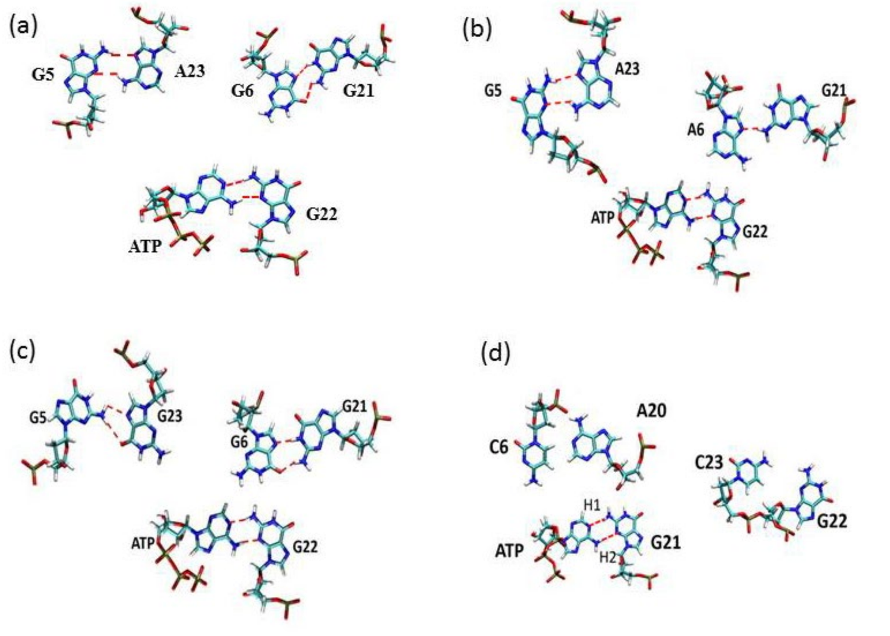

Fig. S4 Average structures of the significant non-Watson-Crick base pairs in the aptamers and their complexes with ATP, which preserve the stability of the active site. (a) wt aptamer, (b) mt 1 apt., (c) mt 2 apt. and (d) mt 3 apt. The hydrogen bonds are indicated with dashed lines.

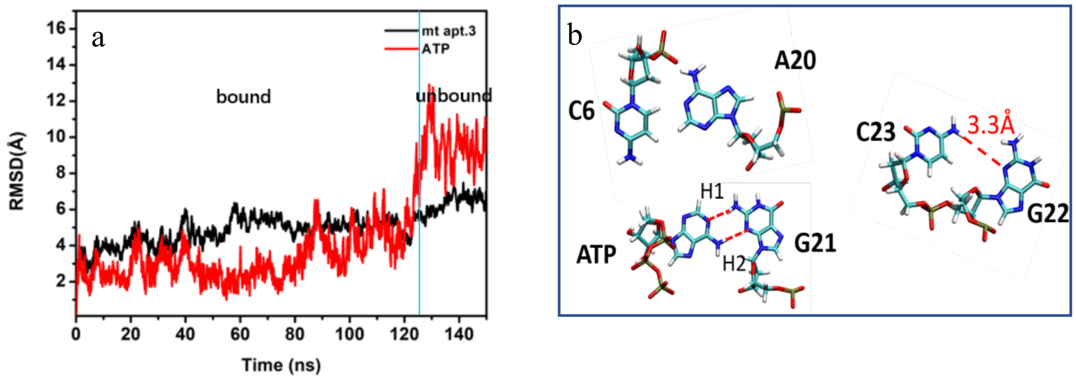

Fig. S5 a) The RMSD of mt 3 aptamer during the full simulation time. b) Base interactions in the binding site in the ATP-mt apt. 3 complex. Data shown for the most stable of the two replicas.

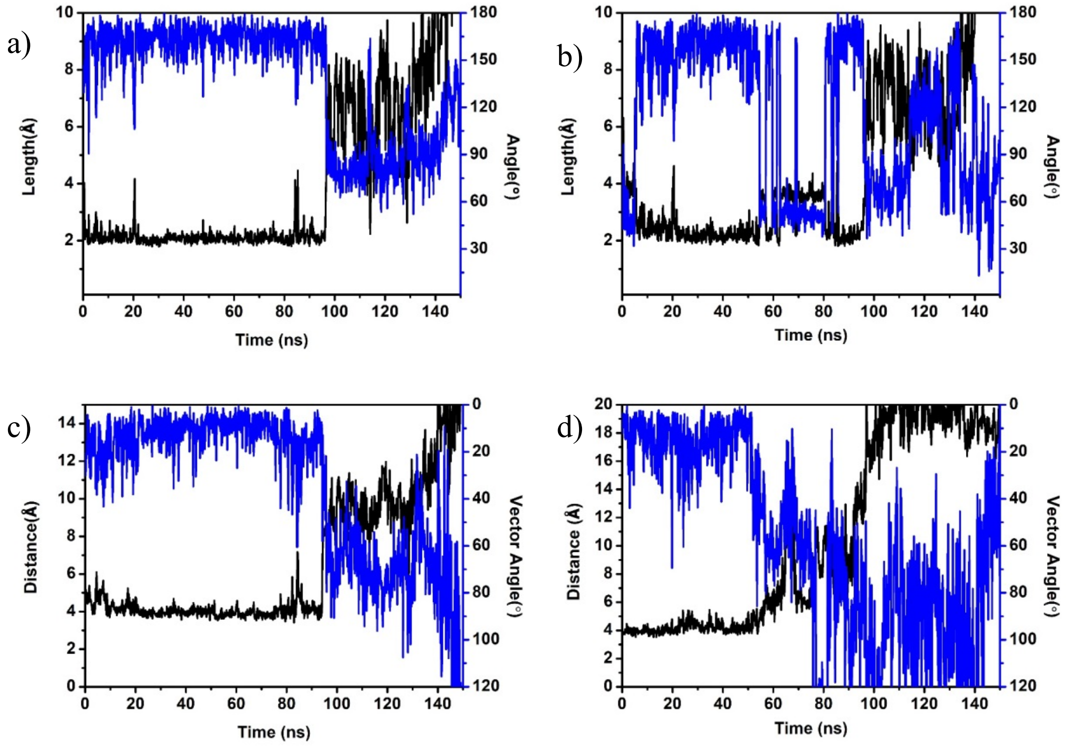

Fig. S6 Data for the hydrogen bonds (a) H1 and (b) H2, respectively in the most stable of the two replicas of the mt aptamer 3 – ATP complex. Hydrogen bond lengths in black (left scale) and angles in blue (right scale). Distance (black, left scale) and vector angle (blue, right scale) between the ring systems of (c) C6 and ATP and (d) ATP and C23. Distances and angles as defined in the text. Data shown for the most stable of the two replicas.

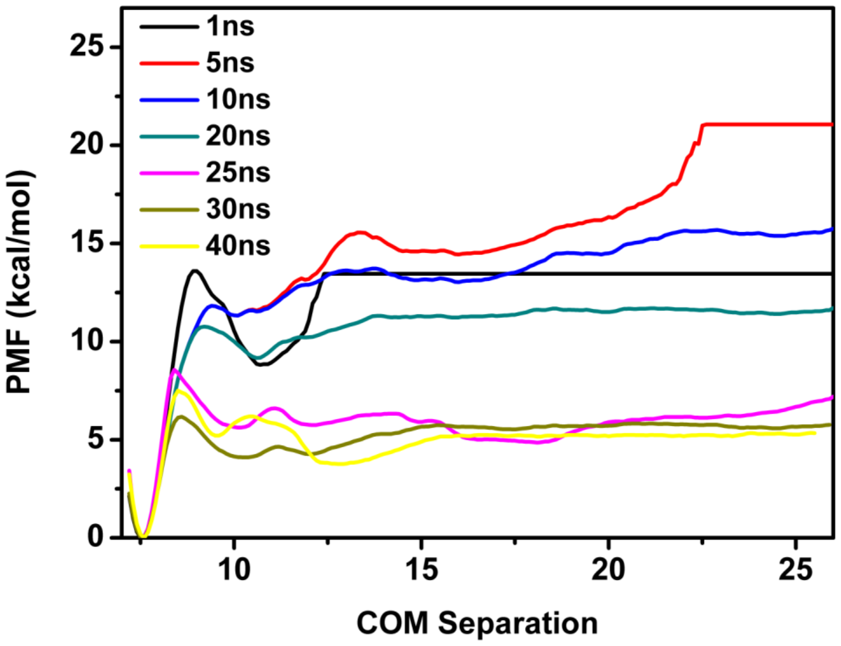

Figure S7. Free energy profiles as a function of the CV COM distance between G22 and ATP in the wt aptamer – ATP complex for different dissociation times, to assess the convergence of the eABF-meta simulations.

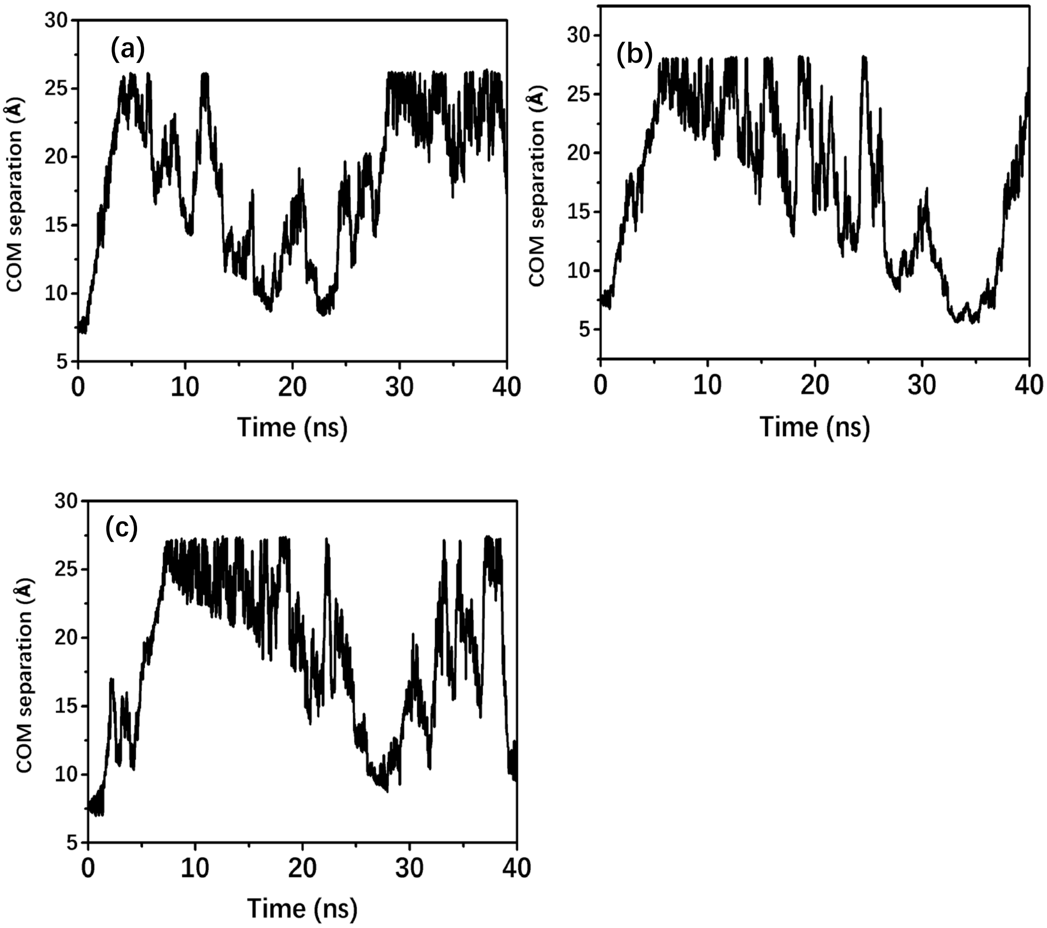

Figure S8. Time evaluation plot of the examined CV of the meta-eABF simulations of the aptamer complexes. (a) Wt aptamer - ATP complex. (b) Mt aptamer 1 - ATP complex. (c) Mt aptamer 2 - ATP complex. The COM data show that a sufficient sampling space of the binding and unbinding processes of ATP is obtained during 40ns.

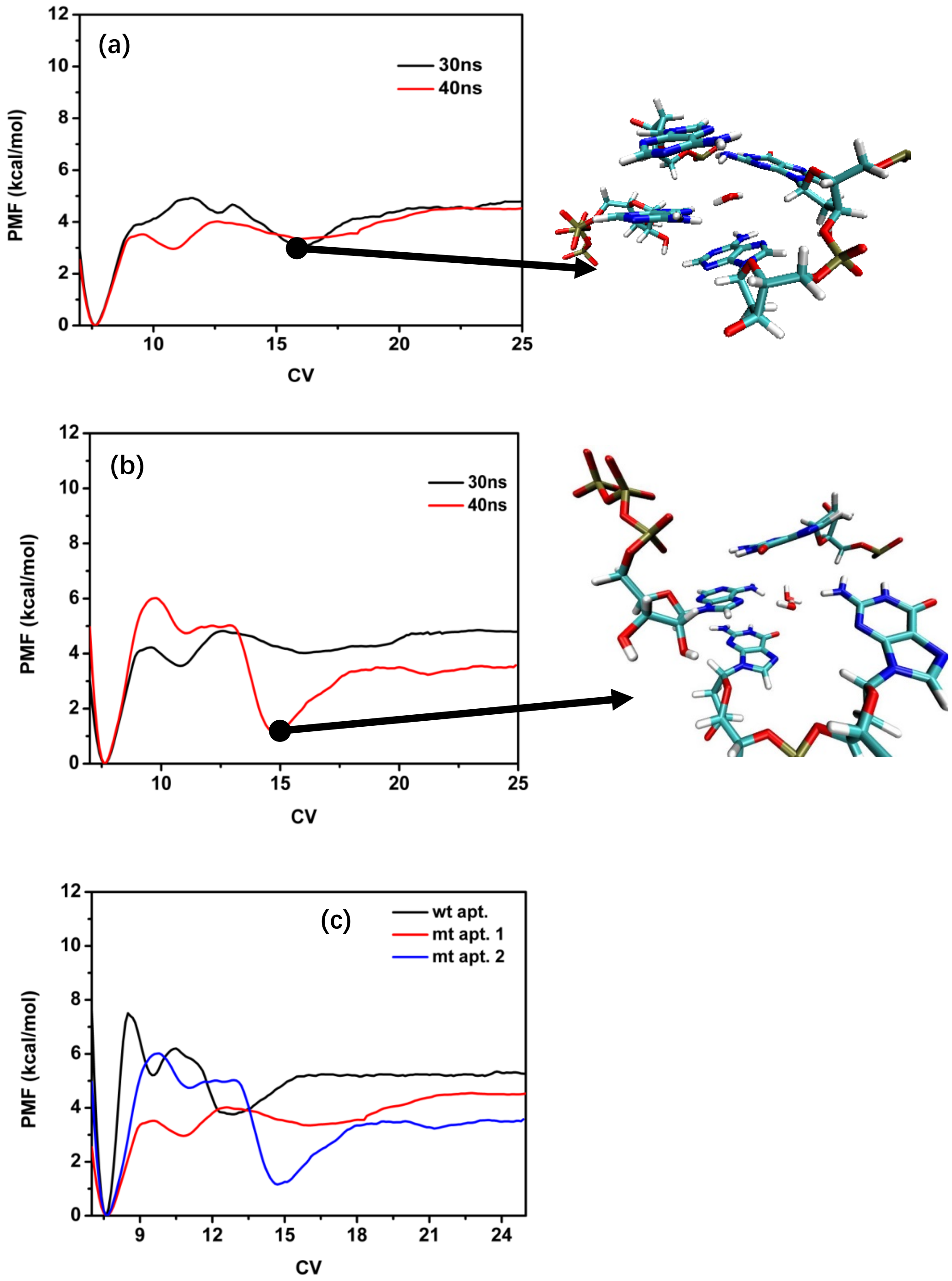

Figure S9. PMF curves for the separation of ATP and (a) mt aptamer 1 and (b) mt aptamer 2, at different reaction time. (c) Free energy curves as function of the center of mass separation during 40 ns dissociation simulations, comparing wt and the two mt aptamer - ATP complexes.
